# The Use of Trimodality Treatment in Patients With Locally Advanced Oesophageal Squamous Cell Carcinoma: An Experience From a Large Cancer Centre in Pakistan

**DOI:** 10.7759/cureus.47835

**Published:** 2023-10-27

**Authors:** Muhammad Fawad Ul Qamar, Huma Hanif, Irfan Haider, Nadia Khaleeq, Dawood Misbah, Yasir Inam, Maheen Anjum

**Affiliations:** 1 Radiation Oncology, Shaukat Khanum Memorial Cancer Hospital and Research Centre, Peshawar, PAK; 2 Medical Oncology, St James's Hospital, Dublin, IRL; 3 Community Dentistry, Khyber Medical University, Peshawar, PAK; 4 Medical Oncology, Mater Private Hospital, Dublin, IRL

**Keywords:** disease free survival (dfs), chemoradiotherapy (chemo-rt), neoadjuvant chemo radiation, concomitant chemo-radiation therapy, overall survival (os), esophageal squamous cell carcinoma (scc), pathologic complete response, oesophageal squamous cell carcinoma

## Abstract

Introduction

Esophageal cancer is one of the most common cancers worldwide. Neoadjuvant chemoradiotherapy followed by surgery is the standard treatment for locally advanced squamous cell carcinoma (SCC). Pathological complete response (pCR) after surgery is associated with better outcomes in terms of overall survival and disease-free survival. We aim to determine the effectiveness of neoadjuvant chemoradiotherapy in patients with locally advanced SCC at our institute, the largest purpose-built cancer center in Pakistan. We also aim to identify various factors influencing pCR, such as chemotherapy regimen, total radiation dose, clinical stage at presentation, and gender.

Materials and methods

This is a retrospective review of all patients with esophageal SCC presented between January 2019 and 2021 to the institute for treatment. Patients received neoadjuvant chemoradiotherapy (nCRT) as per the CROSS trial protocol, followed by surgery. We assessed the pCR rate. Statistical analyses were performed using IBM SPSS Statistics for Windows, Version 22 (Released 2013; IBM Corp., Armonk, New York). pCR was studied alongside associated factors such as age, gender, stage of disease, chemotherapy regimen, and total dose of radiotherapy. A p-value of <0.05 was considered statistically significant. The chi-square test was used to compare categorical variables. Univariate and multivariate logistic regression was employed to evaluate factors affecting pCR.

Results

A total of 218 patients were included in the study. pCR was achieved in 64.2% of the patients. The female gender was associated with better outcomes, as 70.4% (n=81) of female patients achieved a complete pathological response, compared to 57.3% (n=59) of males, with a p-value of 0.03. On univariate analysis, the complete pathological response was 69.6% (n=94) in the age group of 45 years and below, whereas it was 55.4% (n=46) in the age group above 45 years, with a p-value of 0.024. Though statistically insignificant, outcomes were slightly better for those with node-negative disease, as 67.2% (n=41) achieved complete pathological response compared to those with node-positive disease at 63.1% (n=99). Univariate logistic regression analysis identified gender (p=0.044, OR=1.77, 95% CI: 1.016-3.108) and age group (p=0.034, OR=1.844, 95% CI: 1.046-3.252) as significantly associated with pCR. Female patients were 77% more likely to achieve pCR compared to male patients (OR=1.77, 95% CI: 1.016-3.108). Younger patients (≤45 years) were 84.4% more likely to achieve pCR compared to the older age group (OR=1.844, 95% CI: 1.046-3.252). However, these did not maintain significance in multivariate logistic regression analysis.

Conclusion

Our study indicated a high rate of pCR with nCRT in patients with esophageal SCC compared to other studies. The achievement of pCR was higher among females and younger patients, which was statistically significant on univariate logistic regression analysis. Our study also concluded that a higher dose of RT (50Gy/25#) is not superior to a lower dose (45Gy/25#) in terms of pCR achievement but was statistically insignificant. Similarly, CARBO/PAC was not superior to CIS/CAP in terms of pCR achievement and was also statistically insignificant.

## Introduction

Esophageal cancer is the eighth most common cancer worldwide in terms of incidence and ranks sixth in causing cancer-related deaths [1,2]. The average 5-year overall survival (OS) rate is 20%. However, for localized disease, it is around 46% and declines with regional and distant metastasis [3]. Esophageal cancer has two major types of histology: squamous cell carcinoma (SCC) and adenocarcinoma (AC). While AC is more common in North America and other regions of Europe, SCC is more common in Asia, Africa, and Southern Europe [4]. Due to the lack of early screening, mostly half of the patients are diagnosed with locally advanced diseases [5]. The treatment options vary according to staging. For early-stage (cT1 N0 M0 and low-risk cT1 N0 M0) esophageal cancer, upfront surgery is preferred, irrespective of histology. However, for locally advanced (cT2-4 N1-3 M0 and high-risk cT2 N0 M0) SCC, neoadjuvant chemoradiotherapy (nCRT) followed by esophagectomy is the standard of treatment [6]. Since 1992, when the RTOG 85-01 study demonstrated a significant survival advantage with CCRT (concurrent chemoradiotherapy) and a cisplatin and 5-fluorouracil (Cis/5-FU) regimen compared to radiation alone, Cis/5-FU has been used often in conjunction with radiotherapy for patients with unresectable locally advanced esophageal squamous cell cancer (ESCC) [7].

The CROSS-trial set the benchmark for neoadjuvant CRT followed by surgery for locally advanced non-metastatic esophageal cancers, with an absolute 10-year OS benefit of 13%. Following nCRT, as much as 49% of patients achieved a pathological complete response (pCR) in the SCC group, whereas in the AC group, it was 29%, with a significantly longer median OS of 48.6 months [8]. For those patients who respond poorly to nCRT, the PALACE-1 trial in which preoperative pembrolizumab combined with chemoradiotherapy (PPCT) offered a safe and feasible regimen for treating resectable esophageal SCC. A pathologic complete response rate of 55.6% following PPCT also indicated potential therapeutic efficacy [9]. A pCR is defined as the absence of viable cancer cells in the resected esophagectomy specimens. It is regarded as a prognostic marker along with improved OS and disease-free survival (DFS) in patients achieving a complete pathological response [10].

Since achieving a complete pathological response is associated with improved OS and progression-free survival, in this study, we aim to determine the effectiveness of neoadjuvant CRT in patients with locally advanced SCC in our institute, which is the largest purpose-built cancer center in Pakistan. We also aim to determine various factors that can influence pCR, such as the chemotherapy regimen, total radiation dose, clinical stage at presentation, and gender.

## Materials and methods

Study population

This is a retrospective review of all patients with histologically confirmed esophageal SCC who presented between January 2019 and December 2021 to the institute for treatment. The population included people of all age groups and were predominantly from the Northwest of Pakistan and Afghanistan.

Inclusion criteria

Histologically confirmed esophageal SCC of stage cT2-4 N1-3 M0 and high-risk poorly differentiated cT2 N0 in all age groups were included. Only patients with a performance status of ECOG 0-2 were included.

Exclusion criteria

Patients with non-SCC histology, non-compliance with treatment, metastatic disease, advanced inoperable esophageal cancer such as cT4bN2/3, and a previous or current diagnosis of other cancers were excluded.

Treatment protocol

These patients received nCRT followed by surgery. We assessed the pCR rate. Patients received either nCRT (chemoradiotherapy) with capecitabine plus cisplatin or carboplatin plus paclitaxel. The former consisted of two cycles of intravenous cisplatin at 60 mg/m^2^ on day 1 and oral capecitabine at 825 mg/m^2^ twice daily from days 1 to 14 at three-week intervals. The latter included carboplatin AUC-2 weekly along with paclitaxel at 50 mg/m^2^ weekly.

A Varian Vital Beam machine was used to deliver the RT using the volumetric modulated arc therapy (VMAT, RapidArc) technique. The dose of RT was 45 Gray (Gy) or 50 Gray completed in 25 fractions over 5 weeks (1.8 to 2 Gy per fraction). This was followed by laparoscopic minimally invasive esophagectomy or trans-hiatal esophagectomy.

pCR was defined as a complete response to nCRT followed by no detection of cancer on histopathology of the surgical specimen post esophagectomy.

Ethical approval

Since this is a retrospective study based purely on hospital records, informed consent is not required for this study. Moreover, the data was fully anonymized to avoid the breach of patients’ confidentiality. The study was approved by the institutional review board (IRB) of Shaukat Khanum Memorial Hospital and Research Centre.

Statistical analysis

Statistical analyses were performed using IBM SPSS Statistics for Windows, Version 22 (Released 2013; IBM Corp., Armonk, New York). pCR was studied along with its associated factors such as age, gender, stage of disease, chemo regimen, and the total dose of radiotherapy, with p < 0.05 considered statistically significant. The chi-square test was used to compare categorical variables. Univariate and multivariate logistic regression was used to identify the association of pCR with various variables. The pre-op T stage and pre-op N stage were not included in logistic regression studies due to a wide variance in sample distribution. Only statistically significant variables on univariate logistic regression were included in multivariate analysis.

## Results

**Figure 1:**
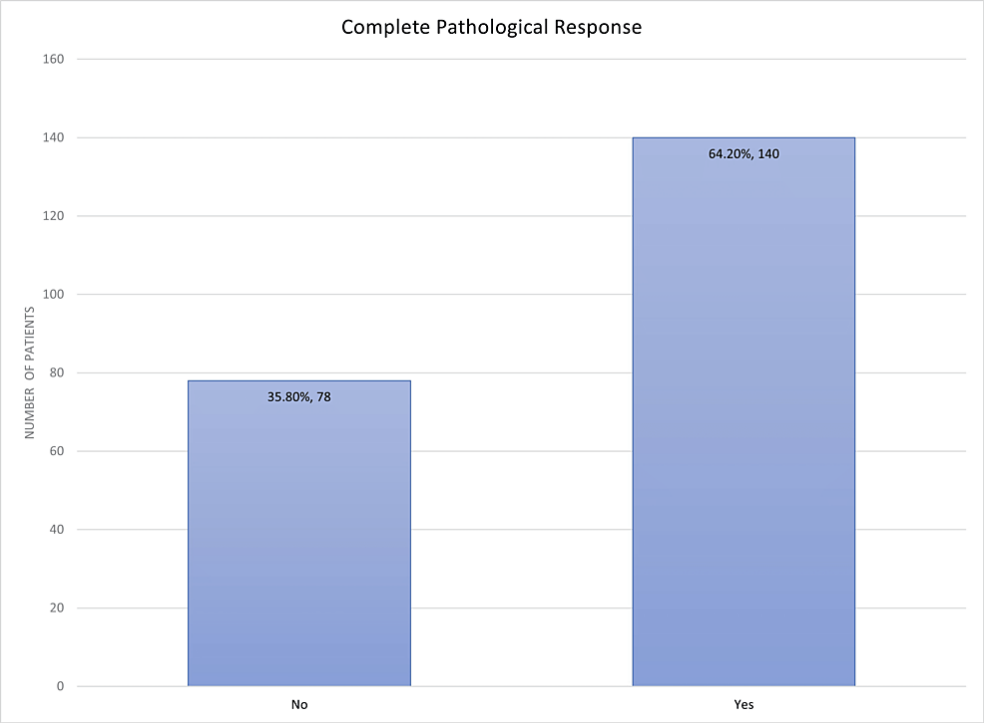
Complete pathological response

A total of 218 patients were included in the study. Complete pathological response was achieved in 64.2% (n=140) of the patients (Figure 1).

**Figure 2:**
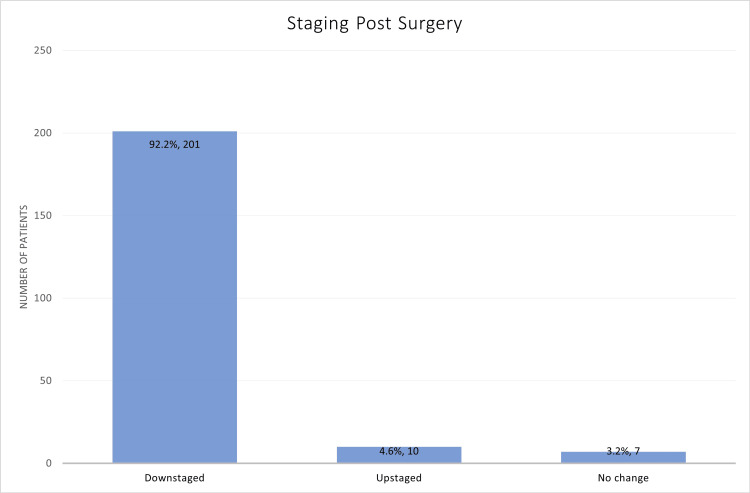
Downstaging versus upstaging after surgery

Most patients (92.2%, n=201) had downstaging after chemoradiotherapy and surgery. Only 4.6% (N=10) of the patients had upstaging after surgery, whereas 3.2% (n=7) of patients had no change (Figure 2).

The mean age was 44 ± 8.93 years. The majority of the patients were female (52.8%, n=115). Most of the patients (89.4%, n=195) had T4 disease on presentation. With regards to nodal status, 54.6% (n=119) of the patients had an N1 disease on presentation (Table 1).

**Table 1:** Demographics

Variables		Frequency	Percentage
Gender	Male	103	47.2
	Female	115	52.8
Pre-op - T stage	T1	2	0.9
	T2	4	1.8
	T3	195	89.4
	T4	17	7.8
Post-op - N stage	N0	193	88.5
	N1	21	9.6
	N2	3	1.4
	N3	1	0.5
Post-op - T stage	T0	149	68.3
	T1	11	5.0
	T2	25	11.5
	T3	32	14.7
	T4	1	0.5
Pre-op - N stage	N0	61	28.0
	N1	119	54.6
	N2	36	16.5
	N3	2	0.9
Age in years (mean + SD)		44 + 8.93	

Female gender was associated with a better outcome as 70.4% (n=81) of the female patients achieved a complete pathological response, whereas in males, the percentage was 57.3% (n=59). This was statistically significant with a p-value of 0.03 (Table 2).

The younger age group had a better outcome as a complete pathological response was observed in 69.6% (n=94) of individuals in the age group of 45 and below, whereas in the age group above 45, it was 55.4% (n=46). This was statistically significant with a p-value of 0.024. Although statistically insignificant, the outcome in those having node-negative disease was slightly better, with 67.2% (n=41) of the patients achieving a complete pathological response compared to those with node-positive disease (63.1%, n=99) (Table 2).

There was no significant difference noted between patients receiving 45 Gray in 25 fractions vs. 50 Gray in 25 fractions, with almost similar outcomes in terms of a complete pathological response at 60.2% (n=62) and 67.2% (n=78), respectively. Most of the patients (88%, n=192) received concurrent carboplatin and paclitaxel along with radiotherapy. However, no significant difference was noted in terms of a complete pathological response between the carboplatin/paclitaxel group and the cisplatin/capecitabine group, at 64.1% and 65.4%, respectively.

With regards to TNM staging, pCR was highest (100%) among patients with T2 disease; however, there were only four patients. This was followed by T3 with a pCR of 64.6% and T4 with a pCR of 58.8%. The results were statistically insignificant with a p-value of 0.1. pCR was highest (72.2%) among patients with N2 disease followed by N0 (67.2%) and N1 (60.5%). However, the results were statistically insignificant with a p-value of 0.545 (Table 2).

**Table 2:** Factors associated with pCR pCR: pathological complete response, CARBO/PAC: carboplatin/paclitaxel group, CIS/CAP: cisplatin/capecitabine group

Complete pathological response associated with gender					
Gender	pCR	Yes	No	Total	p-Value
	Male	59 (57.3%)	44 (42.7%)	103 (100%)	0.03
	Female	81 (70.4%)	34 (29.6%)	115 (100%)	
	Total	140 (64.2%)	78 (35.8%)	218 (100%)	
Complete pathological response associated with age group					
Age	pCR	Yes	No	Total	p-Value
	Above and equal to 45 years	46 (55.4%)	37 (44.6%)	83 (100%)	0.024
	Below 45 years	94 (69.6%)	41 (30.4%)	135 (100%)	
	Total	140 (64.2%)	78 (35.8%)	218 (100%)	
Complete pathological response associated with nodal status					
Nodal involvement vs non-nodal	pCR	Yes	No	Total	p-Value
	Node positive	99 (63.1%)	58 (36.9%)	157 (100%)	0.34
	Node negative	41 (67.2%)	20 (32.8%)	61 (100%)	
	Total	140 (64.2%)	78 (35.8%)	218 (100%)	
Complete pathological response associated with dose and fractionation of radiation					
Dose and fractions	pCR	Yes	No	Total	p-Value
	45 Gy/25#	62 (60.2%)	41 (39.8%)	103 (100%)	0.151
	50 Gy/25#	78 (67.8%)	37 (32.2%)	115 (100%)	
	Total	140 (64.2%)	78 (35.8%)	218 (100%)	
Complete pathological response associated with the chemotherapy regimen					
Chemo regimen	pCR	Yes	No	Total	p-Value
	CARBO/PAC	123 (64.1%)	69 (35.9%)	192 (100%)	0.5
	CIS/CAP	17 (65.4%)	9 (34.6%)	26 (100%)	
	Total	140 (64.2%)	78 (35.8%)	218 (100%)	
Complete pathological response associated with pre-op T-staging					
Pre-op - T stage	pCR	Yes	No	Total	p-Value
	T1	0 (0%)	2 (100%)	2 (100%)	0.1
	T2	4 (100%)	0 (0%)	4 (100%)	
	T3	126 (64.6%)	69 (35.4%)	195 (100%)	
	T4	10 ( 58.8%)	7 (41.2%)	17 (100%)	
	Total	140 (64.2%)	78 (35.8%)	218 (100%)	
Complete pathological response associated with pre-op N-staging					
Pre-op - N stage	pCR	Yes	No	Total	p-Value
	N0	41 (67.2%)	20 (32.8%)	61	0.545
	N1	72 (60.5%)	47 (39.5%)	119	
	N2	26 (72.2%)	10 (77.8%)	36	
	N3	1 (50%)	1 (50%)	2	
	Total	140 (64.2%)	78 (35.8%)	218 (100%)	

Univariate logistic regression analysis identified gender (p=0.044) and age group (p=0.034) as significantly associated with pCR. Female patients were 77% more likely to achieve pCR compared to males (OR=1.77, 95% CI 1.016-3.108). Patients in the younger age group (>45 years) were 84.4% more likely to achieve pCR compared to the older age group (OR=1.844, 95% CI 1.046-3.252). Only statistically significant variables (p-value <0.05) in univariate analysis were included in multivariate analysis. However, both gender and age did not maintain their significance on multivariate logistic regression (Table 3).

**Table 3:** Univariate and multivariate logistic regression for factors associated with pCR pCR:  pathological complete response, OR: odds ratio

	Univariate			Multivariate		
	OR	CI 95%	p-value	OR	CI 95%	p-value
Gender	1.777	1.016-3.108	0.044	1.671	0.948-2.946	0.076
Age group	1.844	1.046-3.252	0.034	1.738	0.979-3.087	0.059
Chemo regimen	1.06	0.448-2.504	0.895			
Radiation dose	1.394	0.800-2.430	0.241			
Node status	1.201	0.643-2.244	0.566			

## Discussion

nCRT in ESCC can cause tumor downstaging, higher rates of negative resection margins, reduced recurrence rates, and improved survival [11]. The current study is a unique retrospective study that not only evaluates the rate of pCR following nCRT but also assesses the effect of different factors on the achievement of pCR. The current findings in our study revealed that 140 patients (64.2%) had achieved pCR among the 218 patients with ESCC who were enrolled in the study. In other studies that used pCR as a clinical response evaluator, a clinical Chinese study (NEOCRTEC5010) revealed that 43.2% achieved pCR among 185 ESCC patients [12]. Similarly, in another Chinese study, 25.76% of 392 SCC patients achieved pCR [13]. In another Indian retrospective study, the pCR rate was found to be 55.5% among ESCC patients [14]. The CROSS trial, which was a benchmark for nCRT, showed a pCR rate of 49% in the squamous cell cancer group [5]. Similarly, another study done in Iran showed that 40.6% of the patients with SCC achieved pCR [15].

In our study, female gender was associated with a better outcome (p=0.044, OR=1.77, 95% CI 1.016-3.108) as 70.4% of females achieved pCR, whereas in males, the percentage was 57.3%. This is concordant with another study that showed that female gender had better outcomes in achieving pCR, as it was 59% for females and 41% for males [14]. In contrast, the NEOCRTEC5010 study revealed that males achieved pCR more than females, as the male percentage was 82.5% [12].

Regarding age, there was a favorable link in our study between the younger age group (p=0.034, OR= 1.844, 95% CI 1.046- 3.252) and complete pathological response pCR, with 69.6% (n=94) of patients of age group 45 and below having a complete pathological response, whereas only 55.4% (n=46) of patients over 45 achieved pCR. Other studies showed that the mean age for complete pathological response was 52 and 55.43, respectively [12,14]. With regards to nodal involvement, despite being statistically insignificant, results were slightly better in patients with N0 disease: 67.2% (n=41) of these patients achieved pCR as opposed to 63.1% (n=99) of patients with nodal disease. One study showed that 76% of patients with a pathologic complete lymph node regression (LNR) score of 1 had achieved a pCR in the primary tumor [16]. In contrast, another recent study revealed that the pCR rate was lower in node-negative disease at only 43%, while it was achieved in 57% of those with nodal involvement [14].

We examined the effect of the dose of radiation on achieving pCR. Our study indicated that there was no discernible difference between the group receiving 45 Gy or 50 Gy in 25 fractions. A recent study showed that a complete pathological response was noted in 20% of patients receiving 36 Gy radiotherapy and 45% of patients receiving 50 Gy radiotherapy [17]. Another study on 214 patients found that pCR was obtained in 13% of patients treated with 30-30.6 Gy, 38% of patients treated with 39.6-40 Gy, and 67% of patients treated with 44-45 Gy. They concluded that the dose of radiation correlates significantly with the likelihood of achieving a pCR in SCC patients [18]. In the same context, another study noticed that 42.9% of patients who received ≤45 Gy achieved pCR while in patients who received more than 45 Gy, the pCR rate was 30.6% [19]. This points towards a better response with higher doses of radiation but at the expense of slightly worse side effects. However, 45-50 Gy is the standard dose, and no significant differences have been noted in terms of side effects or response.

Our study also indicated that there was no statistically significant difference in achieving pCR with different concurrent chemotherapy regimens. One study showed that neoadjuvant CRT with carboplatin and paclitaxel doublet is associated with a better survival outcome, higher surgical resection rate, and better safety profiles than cisplatin and capecitabine in patients with locally advanced esophageal SCC. However, pCR was not examined [20].

Our study showed that after nCRT and surgery, most patients (92.2%, n=201) had their tumor downstaged, compared to 3.2% (n=7) of patients who had no change, and only 4.6% (n=10) of patients had upstaging after surgery. Another study investigated the effects of nCRT on TNM stage and its prognostic significance and found that 68% of the patients being downstaged [21].

While this study provides valuable insights into pCR attainment in SCC patients, it is important to note that there were some limitations regarding the detection of OS and DFS in patients with ESCC due to its importance for prognostication, treatment decision-making, patient counseling, and clinical research. These limitations should be addressed in future research. The pCR rate in our study was higher as compared to other studies. Of note, no such studies have been conducted to the best of our knowledge across our patient population. Whether the response rate is higher due to differences in tumor biology or patient population is a conundrum, and perhaps further studies with newer modalities of treatment along with outcomes in terms of OS and DFS could be crucial.

## Conclusions

Our study indicated a high rate of pCR with nCRT in patients with esophageal SCC as compared to other studies. The achievement of pCR was higher amongst females and younger patients which was statistically significant on univariate logistic regression analysis. Our study also concluded that a higher dose of RT (50 Gy/25#) is not superior to a lower dose of RT (45 Gy/25#) in terms of achievement of pCR but was statistically insignificant. Similarly, CARBO/PAC was not superior to CIS/CAP in regard to the achievement of pCR but was statistically insignificant as well.

## References

[1] Liu CQ, Ma YL, Qin Q, Wang PH, Luo Y, Xu PF, Cui Y (2023). Epidemiology of esophageal cancer in 2020 and projections to 2030 and 2040. Thorac Cancer.

[2] Domper Arnal MJ, Ferrández Arenas Á, Lanas Arbeloa Á (2015). Esophageal cancer: risk factors, screening and endoscopic treatment in Western and Eastern countries. World J Gastroenterol.

[3] (2023). Key statistics for esophageal cancer. https://www.cancer.org/cancer/types/esophagus-cancer/about/key-statistics.html.

[4] Huang FL, Yu SJ (2018). Esophageal cancer: risk factors, genetic association, and treatment. Asian J Surg.

[5] van Hagen P, Hulshof MC, van Lanschot JJ (2012). Preoperative chemoradiotherapy for esophageal or junctional cancer. N Engl J Med.

[6] Obermannová R, Alsina M, Cervantes A (2022). Oesophageal cancer: ESMO Clinical Practice Guideline for diagnosis, treatment and follow-up. Ann Oncol.

[7] Cooper JS, Guo MD, Herskovic A (1999). Chemoradiotherapy of locally advanced esophageal cancer: long-term follow-up of a prospective randomized trial (RTOG 85-01). Radiation Therapy Oncology Group. JAMA.

[8] Eyck BM, van Lanschot JJ, Hulshof MC (2021). Ten-year outcome of neoadjuvant chemoradiotherapy plus surgery for esophageal cancer: the randomized controlled CROSS trial. J Clin Oncol.

[9] Zheng Y, Li C, Yu B, Zhao S, Li J, Chen X, Li H (2022). Preoperative pembrolizumab combined with chemoradiotherapy for esophageal squamous cell carcinoma: trial design. JTCVS Open.

[10] Soror T, Kho G, Zhao KL, Ismail M, Badakhshi H (2018). Impact of pathological complete response following neoadjuvant chemoradiotherapy in esophageal cancer. J Thorac Dis.

[11] Jang R, Darling G, Wong RK (2015). Multimodality approaches for the curative treatment of esophageal cancer. J Natl Compr Canc Netw.

[12] Shen J, Kong M, Yang H (2021). Pathological complete response after neoadjuvant treatment determines survival in esophageal squamous cell carcinoma patients (NEOCRTEC5010). Ann Transl Med.

[13] Chao YK, Chang HK, Tseng CK, Liu YH, Wen YW (2017). Development of a nomogram for the prediction of pathological complete response after neoadjuvant chemoradiotherapy in patients with esophageal squamous cell carcinoma. Dis Esophagus.

[14] Nusrath S, Thammineedi SR, Raju KV (2023). Factors associated with pathologic complete response following neoadjuvant chemoradiation and esophagectomy for carcinoma of esophagus and gastroesophageal junction. J Surg Oncol.

[15] Taghizadeh Kermani A, Ghanbarzadeh R, Joudi Mashhad M, Javadinia SA, Emadi Torghabeh A (2022). Predictive value of endoscopic observations and biopsy after neoadjuvant chemoradiotherapy in assessing the pathologic complete response of patients with esophageal squamous cell carcinoma. Front Oncol.

[16] Hsu PK, Yeh YC, Chien LI, Huang CS, Hsu HS (2021). Clinicopathological significance of pathologic complete lymph node regression after neoadjuvant chemoradiotherapy in esophageal squamous cell carcinoma. Ann Surg Oncol.

[17] Lo CM, Wang YM, Chen YH, Fang FM, Huang SC, Lu HI, Li SH (2021). The impact of radiotherapy dose in patients with locally advanced esophageal squamous cell carcinoma receiving preoperative chemoradiotherapy. Curr Oncol.

[18] Ordu AD, Nieder C, Geinitz H (2014). Association between radiation dose and pathological complete response after preoperative radiochemotherapy in esophageal squamous cell cancer. Anticancer Res.

[19] Yang Y, Xu X, Zhou X (2020). Impact of radiation dose on survival for esophageal squamous cell carcinoma treated with neoadjuvant chemoradiotherapy. Front Oncol.

[20] Su PH, Hsueh SW, Tseng CK (2021). Paclitaxel and carboplatin versus cisplatin and 5-fluorouracil in concurrent chemoradiotherapy in patients with esophageal cancer. In Vivo.

[21] Hamai Y, Hihara J, Emi M (2017). Effects of neoadjuvant chemoradiotherapy on pathological TNM stage and their prognostic significance for surgically-treated esophageal squamous cell carcinoma. Anticancer Res.

